# Plasmon-photon conversion to near-infrared emission from Yb^3+^: (Au/Ag-nanoparticles) in tungsten-tellurite glasses

**DOI:** 10.1038/srep18464

**Published:** 2016-01-04

**Authors:** V. A. G. Rivera, Yannick Ledemi, Marcelo A. Pereira-da-Silva, Younes Messaddeq, Euclydes Marega Jr

**Affiliations:** 1 Instituto de Física de São Carlos, Universidade de São Paulo (USP), São Carlos (SP), 13560-970, Brazil; 2 Centro Universitário Central Paulista (UNICEP), São Carlos (SP), 13563-470, Brazil; 3 Center for Optics, Photonics and Laser (COPL), Université Laval, Quebec (Qc), G1V 0A6, Canada

## Abstract

This manuscript reports on the interaction between ^2^F_5/2_→^2^F_7/2_ radiative transition from Yb^3+^ ions and localized surface plasmon resonance (from gold/silver nanoparticles) in a tungsten-tellurite glass. Such an interaction, similar to the down-conversion process, results in the Yb^3+^ emission in the near-infrared region via resonant and non-resonant energy transfers. We associated such effects with the dynamic coupling described by the variations generated by the Hamiltonian *H*_*DC*_ in either the oscillator strength, or the local crystal field, *i.e.* the line shape changes in the emission band. Here, the Yb^3+^ ions emission is achieved through plasmon-photon coupling, observable as an enhancement or quenching in the luminescence spectra. Metallic nanoparticles have light-collecting capability in the visible spectrum and can accumulate almost all the photon energy on a nanoscale, which enable the excitation and emission of the Yb^3+^ ions in the near-infrared region. This plasmon-photon conversion was evaluated from the cavity’s quality factor (Q) and the coupling (g) between the nanoparticles and the Yb^3+^ ions. We have found samples of low-quality cavities and strong coupling between the nanoparticles and the Yb^3+^ ions. Our research can be extended towards the understanding of new plasmon-photon converters obtained from interactions between rare-earth ions and localized surface plasmon resonance.

Plasmonics has played a revolutionary role in the quantum-photon interaction and opened a wide range of practical applications that involve the manipulation of light on a nanoscale. However, issues such as decoherence due to dissipative effects^1^, photon-plasmon conversion^2^,^3^, and enhancement of the luminescence intensity^4^,^5^,^6^ still remain open. In the two latter cases, the question is: what are the mechanisms responsible for those effects? The two possible answers are (i) energy transfer, or (ii) local field enhancement in the quantum-emitter near the metallic nanostructure^3^. Therefore, the distinction between the different mechanisms still constitutes a challenge. Either energy transfer or local field enhancement causes a significant alteration in the local density of optical states (LDOS) of the quantum-emitter, which results in an enhancement/quenching in the emission intensity. This quantum system (quantum-emitter and metallic nanostructure) produces a photon-plasmon interaction, which can be investigated as a weak or strong coupling. Weak coupling is studied within the perturbation theory and its characteristic features are changes in the atomic/molecule decay rates (*e.g.* Purcell effect^7^ and Forster energy transfer^8^). Strong coupling is characterized by a reversible exchange of energy that produces an energy level splitting (Rabi oscillations^9^), where the dynamic behaviour of the coupling is governed by two parameters, namely coupling strength (proportional to the electromagnetic field strength) and detuning (field far away from resonance).

Frequency up/down-conversion has long been a subject of interest in photonic applications^10^,^11^,^12^, since the light signal can be converted from a frequency (wavelength) to another one in a nonlinear-optical medium. The near-infrared light can be converted to light in the visible range (or *vice versa*) and efficiently detected by commercially available detectors. Nonlinear-optical materials show a variety of frequency conversion mechanisms with a wide range of applications to photonics, information, medical, industrial, or military technologies. Among these materials, one can distinguish specialty glasses as chalcogenide or tellurite glasses. In general, glasses are known for their ease of fabrication, chemical composition flexibility and dopants solubility (*e.g.* rare-earth ions (REI)). Among the oxide glasses, tungsten-tellurite glasses (TTG) have shown several advantages, such as wide transmission window (0.4–5.0 μm), good thermal and mechanical stability, intermediate cut-off phonon energy (900–950 cm^−1^), large solubility of REI, and particularly high linear and nonlinear refractive indices (*e.g.* 1.8–2.3 and 20–50 × 10^−20^ m^2^/W, respectively)^13^,^14^,^15^,^16^,^17^. Such a property enables nonlinear effects like second/third harmonic generation, parametric oscillation, and four-wave mixing. Glasses doped with Yb^3+^ ions show efficient emission around 980 nm and match almost 60% of the commercial InGaAs laser diode^18^. Yb^3+^ has a 4f^13^ electronic configuration with two manifolds^2^F_7/2_ and ^2^F_5/2_, *i.e.* it is a two-level quantum emitter whose concentration quenching and multiphonon relaxation should not affect its lasing action^19^. Besides, Yb^3+^ ion is also a sensitizer of energy transfer for infrared-to-visible up-conversion and infrared lasers^10^,^20^.

In this scenario, an accurate control over the emission properties of the Yb^3+^ ion is crucial for the design of novel photonic nano-devices. For instance, many reports on the modification of the optical properties of REI around metallic nanoparticles (NPs) have focused mostly on the luminescence intensity changes^3^,^9^. Localized surface plasmon resonances (LSPRs) from gold or silver NPs (GNPs or SNPs) can significantly modify the Yb^3+^ excited state by means of a strong/weak coupling regime of REI. Metallic NPs have demonstrated excellent light-harvesting capability in the visible spectrum and can collect almost all the photon energy on a nanometric scale. Here, we demonstrate that GNPs or SNPs embedded in an Yb^3+^ -doped TTG can act as a plasmon-photon converter, emitting light in the near-infrared (NIR) via resonant and non-resonant energy transfer processes similar to down-conversion processes. The strong/weak coupling regime between the metallic NPs and Yb^3+^ ions in TTG has also been studied here and results in an enhanced or quenched emission centred at 980 ± 6 nm (Yb^3+^: ^4^F_5/2_ → ^4^F_7/2_ electronic transition) in presence of GNPs or SNPs, respectively.

## Results

**Figure 1:**
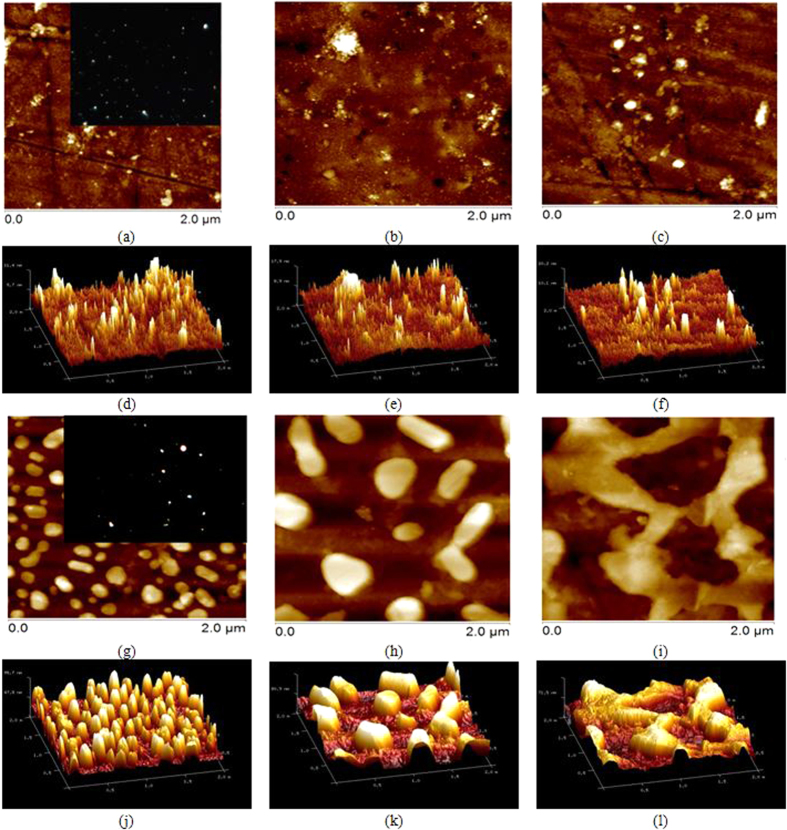
AFM images revealing the 2D/3D morphology of the SNPs for samples (**a**)/(**d**) HM_Y10S, (**b**)/(**e**) HM_Y20S and (**c**)/(**f**) HM_Y30S. 2D/3D AFM images of the GNPs for samples (**g**)/(**j**) HM_Y10G, (**h**)/(**k**) HM_Y20G and (**i**)/(**l**) HM_Y30G. The insets in (**a**,**g**) are the dark-field images of the corresponding samples, both obtained from 10 nm thick deposited films.

For clarity, the glass samples were labelled as HM_YxM, where M represents the noble metal (S or G for silver or gold, respectively) and x (=10, 20 or 30) represents the films thickness (nm). The sample with no Yb^3+^ and metallic film was named HM. The glass transition temperatures T_g_ determined for HM, HM_Y, HM_YxS and HM_YxG are 363 ± 2 °C, 375 ± 2 °C, 378 ± 5 °C and 380 ± 6 °C, respectively. The annealing temperature (=350 °C) used here to nucleate, generate and grow NPs is below the T_g_ of the HM_Y sample. Such a condition was chosen for the reasons discussed by Rivera *et al.*^3^,^20^. Durations of 1 and 3 hours for HM_YxS and HM_YxG samples, respectively, and annealing temperature were adequate to provide enough mobility to the silver or gold atoms, which then nucleate onto and within the glass, forming SNPs or GNPs. A large number of new efficient synthesis routes has been proposed and developed for the production of various plasmonic NPs of different nature, structure, size, and shape^21^. However, in our study, the size and shape of the NPs were achieved by varying the thickness of the metallic films and annealing time (Fig. 1). As reported in the literature, silver ions are very mobile in tellurite glass and tend to aggregate easily, forming then metallic NPs^22^. In contrast to silver ions, gold ions exhibit lower mobility in the same glassy network^20^, which can be confirmed, for instance, by Fig. 1(a,g), where both HM_Y10S and HM_Y10G samples show different surface morphologies. The HM_Y10S sample shows the formation of some SNPs of ~ 20 to 100 nm average lateral size and a glass surface roughness in the 2 to 10 nm range. The HM_Y10G sample shows the formation of a large number of GNPs of ~30 to 190 nm average lateral size, and a glass surface roughness in the 8–25nm range with better defined shape in comparison with the SNPs. Additionally, the inset in Fig. 1(a) is a dark-field image extracted from inside the sample showing the white light scattered from the SNPs. A similar result was found in^20^. The insets in Fig. 1(g) show the scattered green light from the GNPs on the glass surface, but no GNPs formation was detected inside the glass. Due to the difference between metal and glass surfaces, the deposition process is far from the thermodynamic equilibrium conditions and the NPs can be formed in the film/glass interface and/or on the metallic surface. Therefore, various processes, such as adsorption and reemission of ions, surface diffusion, glass diffusion, nucleation and agglomeration^23^ compete for the formation of NPs. The formation and growth of SNPs/GNPs (*i.e.* their size and shape) are affected by the respective sticking coefficient of Ag^0^/Au^0^ ions^24^ during the deposition process and annealing time (Fig. 1(a–c,g–i)). Furthermore, the nucleation of NPs within the TTG depends on the diffusion coefficient of Ag^0^/Au^0^ ions. Such a nucleation process can be evaluated through the surface roughness, where the HM_YxS samples are less rough in comparison with the HM_YxG samples (Fig. 1(d–f,j–l)).

The measured densities are 5.77 ± 0.03 g/cm^3^ for the HM_Y sample, 5.84 ± 0.03 g/cm^3^ for HM_YxS, and 5.90 ± 0.02 g/cm^3^ for HM_YxG. The Yb^3+^ ionic density, *N*_*Yb*_ , was calculated according to the method used in^25^. As the chemical composition of the host matrix and the fabrication route remain unchanged for all the prepared samples, the Yb^3+^ ionic density is assumed to be constant at *N*_*Yb*_ ~9.77 × 10^20^ ions/cm^3^. The density of the samples shows a relation HM_Y < HM_YxS < HM_YxG, indicating that the NPs grown on and/or inside the glass induce an increase of the TTG density. A higher density was measured for the TTG samples containing gold NPs because the gold density is higher than that of silver (19.30 and 10.49 g/cm^3^, respectively).

**Figure 2:**
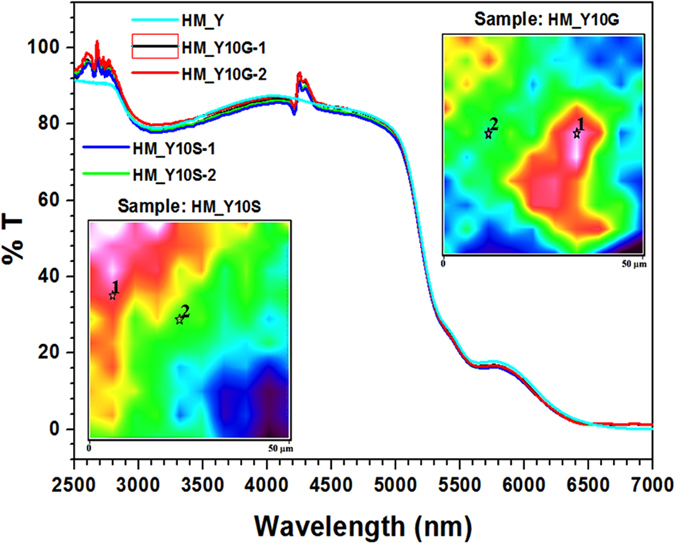
Infared Transmittance spectra of HM_Y (within the spectrometer chamber), HM_Y10G and HM_Y10S (on the sample holder of the microscope). The spectra of HM_Y10(G/S)-(1 and 2) were obtained from points 1 and 2 (see fingerprints images), respectively.

Figure 2 shows the transmittance spectra of the HM_Y, HM_Y10G and HM_Y10S samples. From the fingerprint images, we have chosen two points (1) and (2) of higher and lower NPs concentrations for further micro-transmittance inspection. The obtained curves show practically the same profile. However, the following differences can be noticed: (i) in the 2500 to 2800 nm region, the HM_Y10G and HM_Y10S samples show two additional peaks in comparison with the HM_Y sample, which can be assigned to a red shift of the electric permittivity (ε) oscillations. Such oscillations are usually located in the 1750 to 2200 nm region^26^ but as the refractive index of the tellurite glasses is higher than 1.6 in this range^25^,^27^, such red shift is expected. (ii) In the 2800 to 3950 nm region, the observed broad absorption band is associated with the stretching vibration of OH^-^ groups^28^. The band shape does not change, indicating that NPs did not affect the OH^−^ groups content at points 1 and 2 if compared with the glass (Fig. 2). Nevertheless, subtle variation in the transmittance percentage values can be noticed at ~3250 nm (%T = 78 ± 2%) for the analysed samples, probably because of NPs absorption/scattering on the glass surface (Fig. 1). (iii) In the 4150 to 4400 nm region, a band characteristic to the presence of carbon dioxide (asymmetric stretching vibration modes)^29^ is observed from the spectra recorded on points 1 and 2, which are on the sample surfaces. Such a band is observed only for the HM_Y10S and HM_Y10G samples and may be caused by surface contaminants, such as wax or sandpaper (silicon carbide) residues from the polishing process. Since, possibly, the HM_YxM samples were subjected to an extra thermal treatment for the growth of NPs, the carbon residue on the glass surface can react with the oxygen from the environment and form CO_2_ on the surface of the samples. The CO_2_ band is also routinely observed in the background scan by FTIR spectrometers^30^. Two experimental setups were used for the FTIR measurements. On one hand, the HM_YxM samples spectra were measured on the sample-holder of the microscope, *i.e.* in room environment. On the other hand, the spectrum of HM_Y was measured with the sample placed in the spectrometer chamber which is under continuous nitrogen gas flow. (iv) The IR cut-off wavelength for those samples is ~5200 nm (determined at %T = 50%). (v) As TTGs have tellurium oxide TeO_2_ as a major constituent (70 mol%, acting as the main glass-network former), tungsten oxide WO_3_ (14 mol%, glass-network intermediate acting here as modifier), zinc oxide ZnO (7.5 mol%, acting as glass-network modifier) and germanium oxide GeO_2_ (7.5 mol%, acting as glass-network former), three intrinsic absorption tails of these glasses (Fig. 2) can be expected: (1) one at ~5400 nm, corresponding to the symmetric vibration of WO_4+2_ tetrahedra (930 cm^−1^)^31^, here observed as a second overtone at 1860 cm^−1^ and typically observed for a tungsten oxide-containing glass; (2) one at ~ 5600 nm, corresponding to the stretching vibrations of Ge–O bonds in GeO_4_ tetrahedra (893 cm^−1^) here observed as a second overtone at 1786 cm^−1^ and typically observed around 860 cm^−1^ for a pure GeO_2_ glass;^32^ (3) and another one at ~5900 nm, typical for the tellurite glass^20^,^33^.

**Figure 3:**
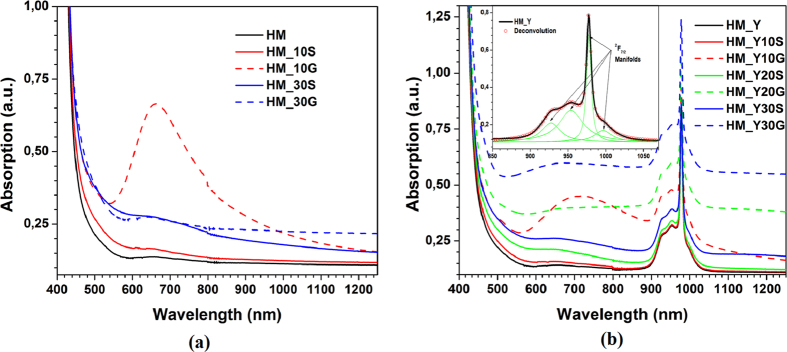
(**a**) Visible-NIR absorption spectra of the samples without Yb^3+^ ions. The samples with GNPs clearly show a resonance band. (**b**) Visible-NIR absorption spectra of the samples doped with Yb^3+^ ions and GNPs or SNPs. The samples with GNPs clearly show a resonance band. Inset: deconvolution of the Yb^3+^ ion absorption band (^2^F_7/2_ level) showing its four Stark splitting.

Figure 3 shows the visible-NIR absorption spectra of the HM and HM_xM samples. The frequency of LSPRs of SNPs and GNPs could be observed and compared with the spectrum of HM, Fig. 3(a). The spectra of HM_10G and HM_30G samples show an intensity maximum centred at 660 and 650 nm, respectively, and the observed broad absorption band is due to the presence of GNPs of different sizes and shapes. Here the striking difference between the two spectra is attributed to the coagulation of the gold films, which results in the nucleation and growth of the NPs in the extra thermal treatment process. The LSPR band could not be detected for HM_10S and HM_30S because its frequency is within the absorption band of HM (<440 nm)^4^,^34^. The absorption spectrum of the HM_YxM samples changes due to scattering losses induced by NPs inside the glass volume and also on their surface. Such optical losses evolve according to the thickness of the films and the material. The visible cut-off wavelength for those samples is ~475 nm, Fig. 3(b). Moreover, we can notice the LSPR band of the HM_YxG samples, the band related to Yb^3+^ (with a maximum peak at 979 nm), and the four splitting (Stark levels = (2J + 1)/2) of the ^2^F_7/2_ level induced by the crystal field (see inset of Fig. 3(b)). The LSPR band does not induce any shift of the peaks position in the HM_YxM samples absorption spectra. Additionally, the spectra of HM_Y10G and HM_Y30G samples show a maximum intensity at 713 and 665 nm, respectively, *i.e.* there is a red-shift in comparison with the spectra of HM_xG samples (660 and 650 nm, respectively). Such a red-shift is due to the refractive index increase which produces a red-shift of the LSPR frequency^3^,^20^,^26^, see Fig. 3(b). Here, the refractive index of the Yb^3+^ -doped tellurite glass is higher than that of the Yb^3+^ -free tellurite glass (of same nominal composition)^16^.

In amorphous materials, a typical short-wavelength absorption edge can be broadly ascribed to one or more of the three following processes: (i) residual below-gap absorption, (ii) Urbach tails^35^ and (iii) interband absorption. Figure 3(b) shows that the fundamental absorption edges are not sharp (a characteristic feature of vitreous materials) and can be described by a power law^36^,^37^. The fundamental absorption edge in the UV or visible regions is due to the existence of extended states observed as an exponential tail (broad/short) and directly related to the conduction and valence bands.

**Figure 4:**
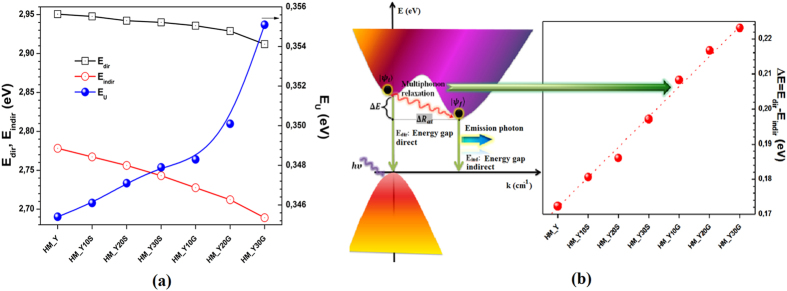
(**a**) Direct, indirect and Urbach energies (E_dir_, E_indir_ and E_U_, respectively) calculated for each studied sample. Lines are guides to the eye. (**b**) Proposed energy band diagram (E_dir_ is an electron-hole recombination that results in photon emission, whereas E_indir_ free electrons have an indirect recombination that results in a low photon emission). Here 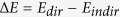 is the multiphonon relaxation energy.

By employing the methodology used in^10^, we calculated and plotted in Fig. 4(a) the direct, indirect and Urbach energies (E_dir_, E_indir_ and E_U_, respectively) for each glass sample of this study. The results show a nonlinear decrease in the E_dir_ and E_indir_ and an increase in their E_U_. Moreover, the values of E_dir_ are higher than the corresponding values of E_indir_. Urbach’s energy refers to the width of the tails of localized states in the forbidden gap of a disordered material. However, in our samples, such an apparent increasing disorder is due to the presence of NPs, *e.g.* E_U_ is lower for the HM_Y sample in comparison with HM_Y30G. The variation in E_U_ with the NPs (size/shape and nature) can be explained by: (i) significant structural changes induced by the NPs within the TTG network, *i.e.* a higher number of defects has been introduced in the glass, and (ii) the scattering produced by the NPs on the TTG surface. Therefore, E_U_ can help in characterizing the structural disorder in amorphous solids (glasses) with embedded NPs, as well as the losses caused by scattering in these materials. Direct and indirect transitions also occur in the glass and involve changes in the crystal momentum, (ћ**k,** ћ is the reduced Planck’s constant and **k** is the wave vector). A photon-phonon or electron-phonon coupling, which is a less likely event in comparison to the direct transition, can be expected from the light-matter interaction. In REI-doped glasses, the energy gap between the excited state and the lower state of REI is often larger than the maximum phonon energy of the host matrix. Therefore, the emission of several phonons is required for the energy conservation (multiphonon relaxation). Tellurite glasses may exhibit various vibrational spectra^16^, thus the rate of multiphonon processes will depend on the glassy matrix composition. In the latter, REI have oxygen and other ions as nearest neighbours^38^ and their vibrations and more distant ions contribute to the fluctuant Stark field, which induces non-radiative transitions. Taking into account the one-dimensional approximation^39^, we will assume that the photon-phonon interaction 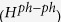 is analogous to the electron-phonon interaction 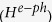
40, since both are nonradiative processes. The coupling matrix element can be written as:


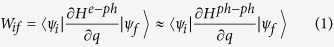


where 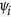 corresponds to the hole in the valence band perturbed by a defect and 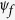
is the localized defect state. In the one-dimensional approximation, an effective vibration corresponds to the displacement of an atom/ion 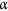 along the direction 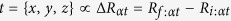. Here, 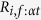 are atomic/ionic coordinates in the equilibrium configuration of the excited (final) and ground (initial) states. The generalized configuration coordinate *q* is defined as:


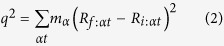


where 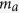 are atomic/ion masses. On the other hand, the Hamiltonian of a REI is^3^:


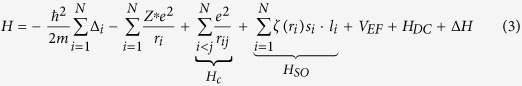


where *N* = 1, …, 14 is the number of 4f electrons, Z^*^e is the screened charge of the nucleus because the closed electron shells have been neglected, *V*_*EF*_ is a potential modelling the interaction between the ion and the electromagnetic field, and 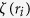 is the spin–orbit coupling function^41^. The Hamiltonian, *H*_*DC*_, is expressed as a function of set 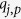 Eigen modes associated with the conduction electron density from NPs(*j*), *i.e.* the collective free oscillations at each resonance frequency *ω*_*p*_ of the NP, as well as their geometric dependence^3^,^20^,^42^. 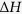 is the perturbation 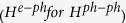 that causes transitions between the eigenstates near 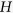 . Here, we consider that the multiphonon relaxation is a nonradiative process in which the electron in a delocalized state is captured to a localized defect state. As a result, two bands arise due to the indirect recombination of an electron in the conduction band, as schematized in Fig. 4(b). The multiphonon relaxation energy, which increases linearly (Fig. 4(b)), can be approximately calculated as:


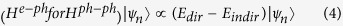


Figure 4(b) also shows a simplified energy band diagram for the glasses studied here, where the lowest minimum of the conduction band and the highest maximum of the valence band lie in different regions of the **k** space. Direct transitions may occur and the indirect transition observed may be associated with transitions from the bottom of the conduction band (*e.g.* second valley) to the top of the valence band.

**Figure 5:**
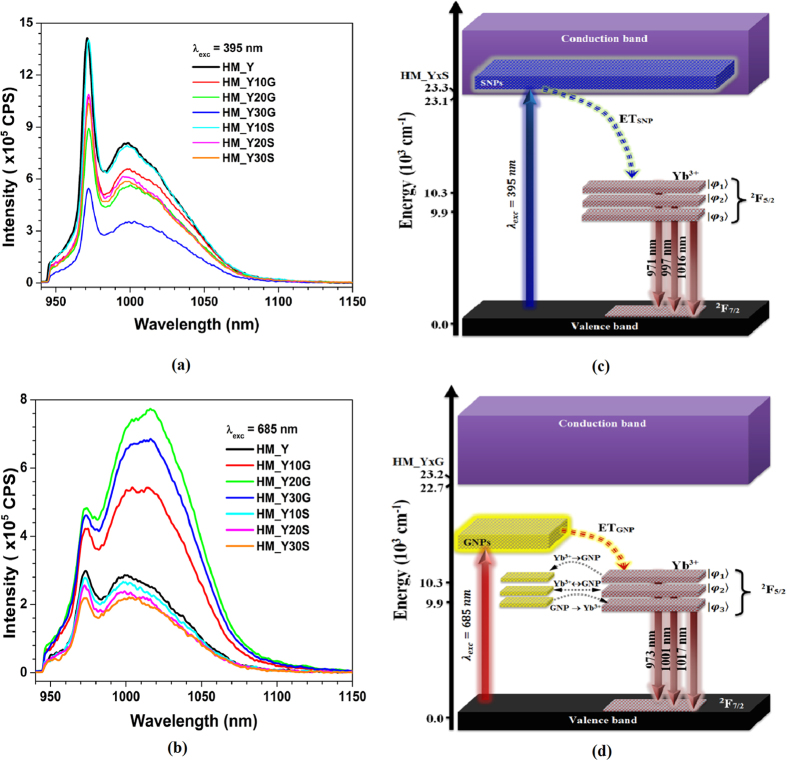
Luminescence spectra of the HM_YxM samples under excitation at (**a**) 395 and (**b**) 685 nm. Simplified energy level diagram of the Yb^3+^ ions with (**c**) SNPs, and (**d**) GNPs with the main transitions that describe the plasmon-photon conversion, as well as the optical band gap for the (**c**) HM_YxS, and (**d**) HM_YxG samples.

**Figure 6:**
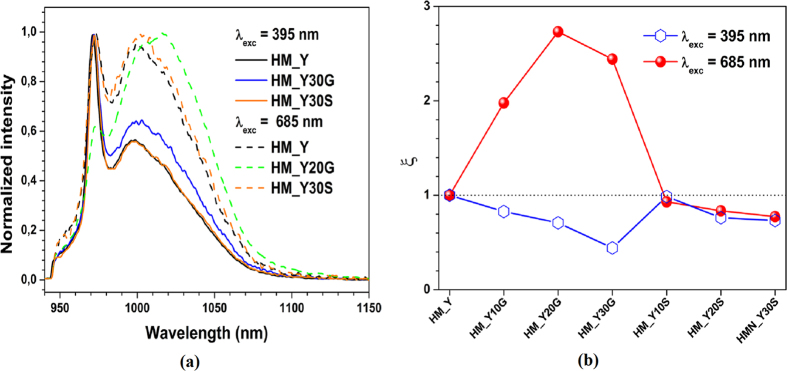
(**a**) Normalized emission intensity of the Yb^3+^ -doped TTG samples containing GNPs or SNPs under 395 and 685 nm excitation. (**b**) Quantum yields ξ of all the studied samples under 395 and 685 nm excitation wavelengths.

Figure 5 shows the luminescence spectra of the glasses in the 925–1150 nm range under 395 and 685 nm xenon lamp excitations. The luminescence properties of the HM_YxM samples were explored by plasmon-photon conversion to near-infrared emission, where the Yb^3+^: ^2^F_5/2_→^2^F_7/2_ transition is clearly identified. On one hand, when the samples were excited with 395 nm light (Fig. 5(a)), the excitation was produced above the optical band gap of the glass, inside and outside the absorption band of the SNPs and GNPS, respectively, as represented in Fig. 5(c,d). The presence of SNPs significantly decreases the Yb^3+^ emission intensity (approximately 36% of HM_Y emission intensity for the HM_Y30S sample), and the presence of GNPs even further decreases (quenching) it (approximately 76% for the HM_30G sample). On the other hand, when the samples were excited with 685 nm light (Fig. 5(b)), the excitation was produced inside and outside the absorption band of the GNPs and SNPs, respectively, as represented in Fig. 5(c,d). The presence of SNPs decreases the Yb^3+^ emission intensity (approximately 37% for the HM_Y30S sample, similarly to the behaviour observed when this sample was excited at 395 nm), while the HM_YxG samples enhanced the Yb^3+^ emission intensity (approximately 61% for the HM_20G sample), as shown in Fig. 5(b). We normalized the emission spectra of the above-mentioned samples under both excitation wavelengths so as to observe changes in the spectral shape, as presented in Fig. 6(a). In comparison with the spectrum of the HM_Y sample, the HM_Y30S spectrum shows no changes in its shape despite the different excitation wavelengths used. The spectra of the HM_Y(30/20)G samples show changes under the above conditions in comparison with that of the HM_Y sample. The results (shape/size of the NPs, absorption and emission spectra) indicate that the HM_YxG (HM_YxS) samples have (have no) an additional band which is in resonance with the^2^F_5/2_ absorption band of the Yb^3+^ ion. Both effects are represented in Fig. 5(c,d). The plasmon-photon conversion of both HM_YxS and HM_YxG samples is shown in Fig. 5(c,d), which also display the lamp excitation wavelength (*λ*_*exc*_), the three energy levels from the manifold Yb^3+^(^2^F_5/2_) 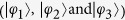, the absorption band of the metallic NPs, the optical band gap and the energy transfer processes (coupling between NPs:Yb^3+^). Below is the operation scheme of this plasmon-photon converter (HM_YxS and HM_YxG):

(i) Under excitation at *λ*_*exc*_ = 395 nm, we populate the conduction band of the samples, which also excites (presumably) the SNPs (for the HM_YxS samples), then an energy transfer (ET_SNP_) from the conduction band can promote electrons to the^2^F_5/2_ state (see Fig. 5(c)). Therefore, we can manipulate the LDOS of the Yb^3+^ via non-resonant energy transfer^3^. A radiative decay initiates the band emission in the near-infrared (^2^F_5/2_→^2^F_7/2_ transition). The spontaneous emission rate is proportional to the LDOS. Nevertheless, such a rate is deficient here, since it does not produce a sufficient coupling between the SNPs:Yb^3+^(Fig. 5(a)) due to detrimental processes as heat generation by Joule effect^4^,^11^,^23^ and multiphonon relaxation (Fig. 4(b)). Both HM_YxG and HM_YxS samples exhibit quenching in the emission intensity, which is higher in HM_YxS in comparison with HM_YxG. This quenching can be explained by the multiphonon relaxation energy, which is higher in HM_YxG than in the HM_YxS samples. Therefore, the quenching of the emission intensity may be assigned to the multiphonon relaxation governed by the photon-phonon and electron-phonon interactions, which damages the coupling between NPs:Yb^3+^. Furthermore, changes are observed in Fig. 5(a) in the spectral shape of the HM_YxG samples in comparison with the HM_Y sample. After the HM_YxG samples have been excited, a non-radiative decay occurs from the conduction band of the glass to the resonance band of the GNPs and produces an energy transfer between the resonance band and the high energy level of the Yb^3+^ 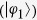 (Fig. 5(d)). The population of this 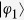 level can migrate to the second and third resonance modes of the GNPs, which can also couple resonantly with the 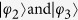 energy levels of Yb^3+^. Therefore, changes in the spectrum line shape and the transition probabilities of those energy levels result from this coupling resonance^4^. The splitting is proportional to the dipole moment of the GNPs. Nevertheless, such an emission process is not efficient due to the energy losses that take place in the conduction band, as mentioned above. Finally, such effects with the dynamic coupling can be described by the variations generated by the Hamiltonian *H*_*DC*_ in either the oscillator strength or the local crystal field, *i.e.* the line shape changes in the emission band.

(ii) Under excitation at *λ*_*exc*_  = 685 nm, the first resonance mode of the GNPs (HM_YxG), after a non-resonant energy transfer (ET_GNP_), promotes electrons to the ^2^F_5/2_ state of Yb^3+^. Such a coupling is efficient (GNPs:Yb^3+^, Fig. 5(b)) and produces an improved spontaneous emission in the near-infrared (^2^F_5/2_→^2^F_7/2_). Nevertheless, the emission intensity of the HM_YxS samples decreases because *λ*_*exc*_ does not produce a population inversion (outside the resonance mode of the SNPs). Subsequently, *λ*_*exc*_ increases the photon-phonon interactions (Figs 4(b) and 5(b)). No change is observed in the spectral line shape of the HM_YxS samples (Fig. 6(a)).

The conversion efficiency (visible to NIR) of these samples can be evaluated through the quantum yields. Because the areas of integrated emission spectra represent the total photon flux, they can be quantitatively compared. Therefore, the quantum yields can be determined as:


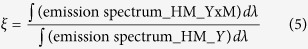


Here we will assume the HM_Y sample has 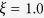, Fig. 6(b). The latter figure shows an overview of the amplitude of 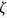 observed in the glasses. ξ depends on the NPs nature, shape and excitation wavelength. Here the HM_Y20G sample exhibits higher conversion efficiency 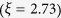 than the other HM_YxM samples. Furthermore, the HM_YxS samples show the same behaviour under 395 and 685 nm excitation (decrease in the emission intensity), which indicates that the SNPs do not increase ξ in the Yb^3+^ emission since they have no resonance band near the Yb^3+^ emission band (900–1100  nm) or other bands (see Fig. 3(a)). When the HM_YxS samples were excited at 395 nm, we related the decrease in the emission intensity to the electron-phonon interaction (in the conduction band); when they were excited at 685 nm, we related the decrease in the emission intensity to the photon-phonon interaction. Therefore, in our initial hypothesis about the one-dimensional approximation, the photon-phonon interaction 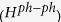 is analogous to the electron-phonon interaction
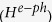
38,39. However, GNPs show short resonance bands that can be coupled with energy levels of Yb^3+^. The HM_YxG samples show higher emission intensities under 685 nm excitation in the NIR and also a quenching under 395 nm excitation if compared with the HM_YxS samples (Figs 5(a) and 6(b)).

The Yb^3+^ radiative emission can be controlled by plasmon-photon conversion by means of a weak or strong electronic coupling, resulting in changes in the LDOS of the^2^F_5/2_ state and an improvement (or not) in the spontaneous emission of Yb^3+^. The REI emission is strongly dependent on the electromagnetic environment (*V*_*EF*_ and *H*_*DC*_). Therefore, this problem can be treated as cavity quantum electrodynamics (CQED). Depending on the nature of this CQED, two main regimes can appear: (i) weak coupling, in which the interaction of Yb^3+^ is basically incoherent and dominated by the damping rates, g ≪ **k**,γ, and (ii) strong coupling whose coherent interaction of Yb^3+^ with the cavity field is described by a coupling constant g, which is a dominant interaction in g ≫ **k**,γ. Different sites can be encountered into the glass network due to the coexistence of glass-formers, intermediates and modifiers usually present as ions. Such sites may be compensated by nearby oxygen atoms (*e.g.* covalently bonded within the glass network) which can compensate for the positive ion nearby since the oxygen atoms are negatively charged. This framework can also be called a CQED, whose quantum-emitter is Yb^3+^ , and can be studied via *V*_*EF*_.

**Figure 7:**
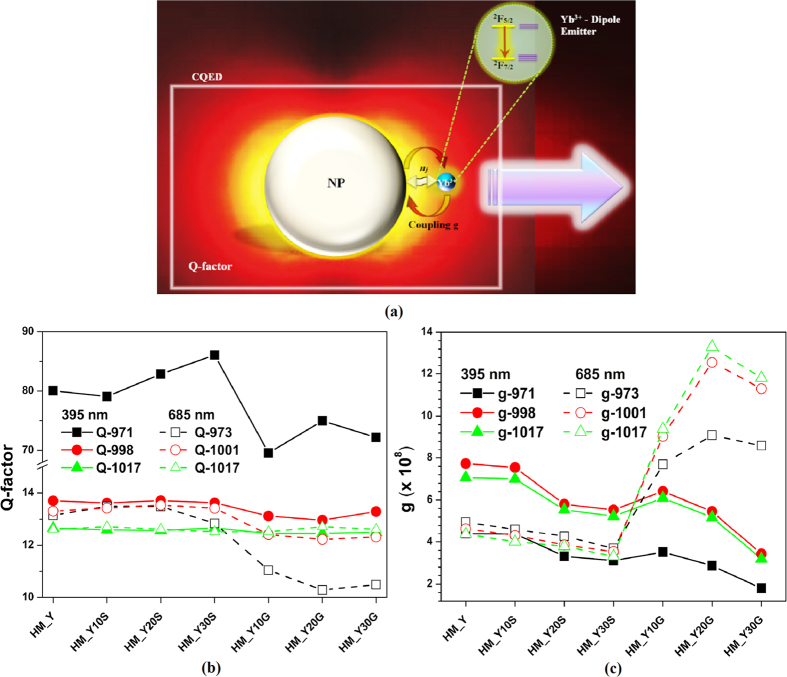
(**a**) Representation of cavity quantum electrodynamics (CQED) formed by a metallic NP and Yb^3+^ ions. (**b**) Q-factor and (**c**) constant coupling g that define the weak or strong coupling of the quantum-plasmonic system in all the studied samples.

Additionally, when a NP is embedded within a REI-doped TTG, the interaction processes in the new CQED can be described by *H*_*DC*_, as represented in Fig. 7(a), and result in a complex coupling^3^,^20^. The intrinsic functionality of this plasmon-photon converter relies in a strong and efficient confinement of the electromagnetic field that can substantially enhance the local field strengths, hence the strength of the interaction between Yb^3+^:NP. The CQED can be evaluated via cavity’s quality factor 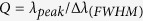. Therefore, the quantum-plasmonic interaction can be estimated by the coupling 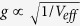
between the electromagnetic field from NPs and the Yb^3+^ ions. This regime involves the emission enhancement/quenching formally defined by the Purcell factor:


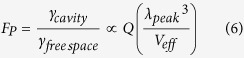


Hence^3^,


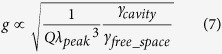


Due to ohmic losses of the NPs, low-quality cavities were obtained (Fig. 7(b)), however, high g values are observed in the HM_YxG samples excited at 685 nm in comparison with the HM_Y sample (Fig. 7(c)). For instance, the HM_Y20G sample shows a higher g than the other samples, owing to its exact resonance (Fig. 5(d)), and a quasi-alignment of the Yb^3+^ ions dipole moment with respect to GNPs. Such properties are responsible for the high conversion efficiency (ξ = 2.73, Fig. 6(b)).

**Figure 8:**
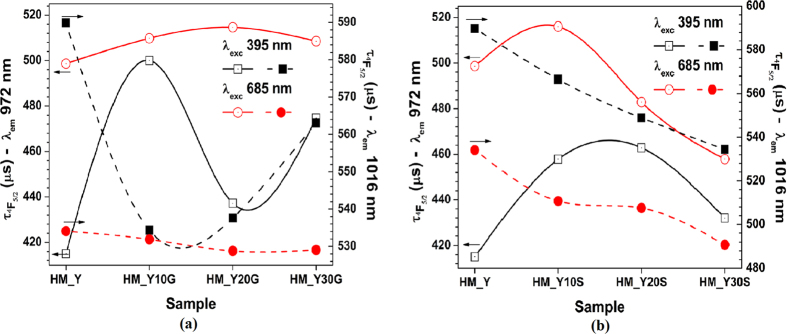
Radiative lifetime measurements at 972 and 1016 nm peaks with λ_exc_ = 395 and 685 nm for the (**a**) HM_YxS and (**b**) HM_YxG samples. Lines are guides to the eye.

Figure 8 presents the lifetime of the ^2^F_5/2_ state. Here, the excitation light (Xe lamp) was set at 395 and 685 nm, *i.e.* the respective SNPs and GNPs absorption bands, and the emission from Yb^3+^ ions was collected at 972 and 1016 nm. These fluorescence lifetimes were affected by the presence of either GNPs or SNPs. Regarding the lifetime dynamics of the HM_YxG samples (under *λ*_*exc*_ = 395 nm) both in the 972 and 1016 nm peaks, we observe a random-increase and a decreasing of the lifetime, respectively (Fig. 8(a)). Nevertheless, when the samples are excited at 685 nm, the lifetime values (at 972 nm) increase, *e.g.* the HM_Y20G sample exhibits a longer lifetime than the other HM_YxM samples. Lifetime dynamics are assigned to the energy transfer process from GNPs to Yb^3+^ ions (Fig. 5(d)). The HM_YxS samples show an increase of lifetime values for the emission at 972 nm under excitation at 395 nm. Here the HM_Y20S sample shows longer lifetime in comparison with the other samples, which is assigned to the energy transfer process from SNPs to Yb^3+^ ions (Fig. 5(c)). However, we observe a lifetime decrease for the emission at 1016 nm due to loss processes within the glass (Fig. 4(b)). Such losses increase when the samples are excited at 685 nm. Moreover, the metal–dipole interaction added an additional channel and provoked a nonradiative decay that depends on the distance between the Yb^3+^ ions and the metallic NPs^3^,^4^. The efficiency of the energy transfer process (above mentioned) depends on many complex parameters, as energy gap (Fig. 5), and competitive non-radiative deactivation processes (Fig. 4).

## Conclusions

Plasmon-photon converters were fabricated from Yb^3+^:(Au/Ag-nanoparticles) in tungsten-tellurite glasses to investigate the dynamic coupling that describes the interaction energy between a quantum emitter (Yb^3+^) and plasmonic nanoparticles. In the studied glasses, metallic nanoparticles increase the Urbach energy, decrease the direct and indirect energies, and increase the electron-phonon and photon-phonon interaction. These glass samples can produce down-conversion emission via energy transfer (changes in the local density of optical states), thus, a resonant coupling between the metallic nanoparticles and the Yb^3+^ ions enhances the emission in the near-infrared region. This enhancement depends on the nature of the NPs and their environment and geometry. We have obtained samples with low-quality cavities and strong coupling between the gold nanoparticles and the Yb^3+^ ions when excited at 685 nm. From a technological point of view, this plasmon-photon converter (with an efficiency of the Yb^3+^ emission improved 2.73 times in the sample HM_Y20G) makes this material a promising candidate to increase the efficiency of solar cells (in the Si-bandgap) via down-conversion process for instance.

## Methods

Tungsten-tellurite glasses (TTGs) of 70TeO_2_–14WO_3_–7.5GeO_2_–7.5ZnO–1Yb_2_O_3_ (mol%) nominal composition were prepared by the conventional melt-casting technique in an induction furnace under low dry oxygen flow (oxidizing conditions)^25^. The glass samples were prepared from high purity starting materials (3N and above). 25 g mixtures were melted in a platinum crucible at 800 °C for 1 hour and annealed near the glass transition temperature for 3 hours. They were then cut into 1.0 × 1.0 cm^2^ pieces and polished up to optical quality with flat parallel surfaces of 0.1 cm thickness so as to minimize the reabsorption issues^43^. The TTGs were then carefully cleaned with distilled water and rinsed with ethanol in an ultrasonic bath (three times, 2 min for each one). The same procedure was strictly followed for each TTG sample to ensure repeatability and control of their optical and thermal properties. Gold or silver thin films of 10, 20 and 30 ± 2 nm thickness (confirmed by a Talystep Rank Taylor Hobson profilometer) were then deposited onto a TTG face for the growth of NPs and subjected to a thermal treatment at 350 °C for 1 and 3 hours for SNPs and GNPs, respectively.

The glass transition temperature (*T*_*g*_) was determined by differential scanning calorimetry (Netzsch DSC 404F3) at a 10 °C/min heating rate in aluminium pans. The density of the samples was determined by the Archimedes method with distilled water as the immersion medium and an electronic densimeter MD-300S (Alfa Mirage). The obtained values correspond to an average of 5 measurements for each glass composition. The nucleation, shape and size distribution of the NPs on the glass were analysed by atomic force microscopy (AFM) using a Digital Instruments Nanoscope 3A multimode microscope. The measurements were performed in tapping mode for the as-prepared samples. The parameters of the structures observed on the glass surfaces were subsequently obtained by processing the AFM images by Digital Instruments software. Fourier transform infrared spectroscopy (FTIR) was carried out on the NPs-TTG surface by a Perkin-Elmer Spectrum Spotlight 400 FTIR imaging microscope system in both imaging and point modes. The FTIR in image mode showed first the area of interest through a CCD camera. A rectangular micro-region was then chosen and the FTIR spectra were recorded at every point in a 5.0 × 5.0 μm^2^ region via a standard liquid-nitrogen-cooled mid-band Mercury Cadmium Telluride (MCT) detector. The typical operating conditions included a 4000–650 cm^−1^ range of 4 cm^−1^ resolution and 32 scans per pixel were averaged to increase the signal-to-noise ratio. The background was recorded with no sample in the sample holder. Dark-field microscopic images were obtained by an optical microscope (Olympus BX53 with a Darkfield 10 × NA0.8 dry condenser) coupled to a digital monochrome camera (Olympus XM10) and using a 75 W Xe lamp as a light source. A transmission dark-field objective (Olympus UPlanSApo 60x/1.2W NA 0.9-NPLan) was used to image the NPs onto the TTGs surface. Absorption spectroscopy measurements were taken at room temperature by a Varian Cary 500Scan UV-VIS-NIR double beam spectrophotometer from 400 to 1250 nm with ± 0.3 nm resolution. Steady-state luminescence spectra were recorded by using a Horiba-Jobin-Yvon Nanolog spectrofluorimeter equipped with a liquid-nitrogen-cooled Symphony II InGaAs near-infrared detector. The measurements were performed under the same conditions for all samples over a 900 to 1250 nm range, with emission acquisitions of 2 s integration time, 10 scans average and 0.5 nm increment steps. The tuneable light source used to excite the samples at 395 and 685 nm is a 450W xenon short-arc lamp (UV to NIR) coupled to a double monochromator with 5 nm slits bandpass. The recorded data were blank-subtracted and corrected for dark count. The lifetime of the^4^F_5/2_ excited state was measured by a Hamamatsu NIR-PMT module detector coupled to the Nanolog system equipped with a Time Correlated Single-Photon Counting (TCSPC) module. The samples were excited by a xenon flash lamp at 395 and 685 nm (1 ms range). DataStation software was used for the data acquisition while DAS6 decay analysis software was used to extract the lifetime values by using a single exponential function fitting. Measurements of both steady-state emission and lifetime were carried out at room temperature and special attention was paid to conduct the optical characterization under identical conditions.

## Additional Information

**How to cite this article**: Rivera, V.A.G. *et al.* Plasmon-photon conversion to near-infrared emission from Yb3^+^: (Au/Ag-nanoparticles) in tungsten-tellurite glasses. *Sci. Rep.* **6**, 18464; doi: 10.1038/srep18464 (2016).
